# Prevalence of active tuberculosis and associated factors among people with chronic psychotic disorders at St. Amanuel Mental Specialized Hospital and Gergesenon Mental Rehabilitation center, Addis Ababa, Ethiopia

**DOI:** 10.1186/s12879-021-06807-z

**Published:** 2021-10-26

**Authors:** Eshetu Temesgen, Yerega Belete, Kibrom Haile, Solomon Ali

**Affiliations:** 1 Department of Microbiology, St. Amanuel Mental Specialized Hospital, Addis Ababa, Ethiopia; 2 Department of Microbiology, Immunology and Parasitology, St. Paul’s Hospital Millennium Medical College, Addis Ababa, Ethiopia

**Keywords:** Active TB, Chronic psychotic disorders, Xpert MTB/RIF assay, Amanuel Mental Specialized Hospital, Gergesenon Mental Rehabilitation Center, SPHMMC

## Abstract

**Background:**

Tuberculosis (TB) is an airborne chronic infectious disease mainly caused by *Mycobacterium tuberculosis complex bacteria*. Currently, about 1.7 billion (26%) of the world’s population are considered to be infected with *M. tuberculosis.* The risk of acquiring tuberculosis is higher on some segments of societies including people with severe mental illness. As a result, World health organization (WHO) strongly recommends screening for tuberculosis in such risk groups and setting.

**Methods:**

A cross-sectional study was conducted to assess the prevalence of active tuberculosis and associated factors among patients with chronic psychotic disorders admitted at St. Amanuel Mental Specialized Hospital and Gergesenon Mental rehabilitation center from February to June, 2020. All admitted patients were screened for any sign of TB as recommended by WHO. Presumptive TB cases were identified. Sputum samples were collected and tested by Xpert MTB/RIF assay. Data analysis was performed using SPSS version 25.0 statistical software and Chi square analysis was used to test the statistical association.

**Results:**

From a total 3600 pschotic patients screened for TB, 250 (6.94%) presumptive tuberculosis cases were detected. From these, 27 (10.8%) were positive by Xpert MTB/RIF assay. Most of the patients were males (68.4%). The mean ± SD age of the participant was 36.5 ± 9.7 years. The overall prevalence of tuberculosis was found to be 750 per 100,000 population. The number of patients per room (p = 0.039) was associated with Xpert MTB/RIF positive active tuberculosis.

**Conclusion:**

The prevalence of active tuberculosis among chronic psychotic patients was high. Number of admitted patients per room was identified as risk factors for Xpert MTB/RIF positive active tuberculosis. Therefore, to control TB transmission in chronic mental health treatment facilities, efforts should be directed to periodic screening for early case detection and improving the number of patients per room.

## Background

Tuberculosis (TB) is an airborne chronic infectious disease mainly caused by *Mycobacterium tuberculosis complex *(*MTBC*) bacteria. It typically affects the lungs (pulmonary TB), but can affect other parts of the body as well (extra pulmonary TB) [1]. The tubercle bacilli are obligate aerobes; grow most successfully in areas of the body with rich in oxygen. It mostly transmitted from one person to the other by the airborne route via aerosols when people with pulmonary TB expel the bacilli while coughing, sneezing, and talking [1, 2]. Tuberculosis remains a major public health problem worldwide and leading cause of morbidity and mortality. Currently, 1.7 billion (26%) of the world’s population are considered to be infected with *Mycobacterium tuberculosis* [3]*.*

In Ethiopia, TB is a major public health problem. The country is still among the 22 high TB burden countries with high number of missed and infectious TB cases in the community. TB is among the top ten causes of admission and deaths in adults in Ethiopia. It is also estimated that Ethiopia had 191,000 new TB cases in 2015. This number ranks Ethiopia 10th globally and 4th in Africa, after Nigeria, South Africa and the Democratic Republic of the Congo. Moreover, Ethiopia is also one of the 27 countries with a high burden of multidrug-resistant tuberculosis (MDR-TB) [4, 5].

A study conducted in Addis Ababa, Ethiopia showed that among 545 TB suspects, 506 (92.7%) of them participated in the study. The prevalence of both pulmonary and extra pulmonary TB was 46.0% (233/506) [6]. Other study conducted in Addis Ababa also showed that *M. tuberculosis* was detected in 1714 participants (13.5%) among a total of 12,685 presumptive tuberculosis patients. Of these MTB cases, 169 cases (9.8%) were confirmed to have rifampicin resistant tuberculosis [7].

Globally different control strategies were implemented for the past two decades to reduce morbidity and mortality due to tuberculosis all over the world. These strategies were, DOTS (directly observed treatment, short-course) strategy of 1995 and the stop TB Strategy of 2006 that aided to reduce by expanded access to high-quality tuberculosis care and address management of all forms of tuberculosis including HIV-associated and drug-resistant tuberculosis*.* Subsequently, these strategies got remarkable achievement in that 37 million lives were saved between 2000 and 2013; a new rapid molecular test, rifampicin resistance was developed and two novel drugs were introduced [8, 9].

Recently, WHO developed the end TB strategy with an overall goal of a 90% reduction in TB incidence and a 95% reduction in TB deaths from 2015 to 2035 by integrated patient-centered care and prevention, bold policies and supportive systems, intensified research and innovation [10].

In spite of all TB control strategies and efforts, tuberculosis is still remained as global public health problem due to weak health systems, determinants such as poverty, under nutrition, migration, diabetes, harmful use of alcohol, tobacco smoking and lack of optimum method for a point-of-care test for rapid diagnosis of disease. In addition, the unmet needs for funding undermine intensification of efforts [9].

Similar to tuberculosis, mental disorders are growing global challenge. People with severe mental illness (SMI) have high risk of developing tuberculosis and other medical illness [1]. One of the greatest challenges has been the fact that the co-morbidity of tuberculosis and severe mental illness was complex, while the vulnerability factors in people with severe mental illness strongly predispose to tuberculosis. Homelessness, HIV, Diabetes, poverty, and alcohol/substance abuse are some of the risk factors that predispose to TB. Hence, it is reasonable to hypothesize that chance of tuberculosis might be higher among patients with mental disorders [1, 9].

According to WHO risk group classification for tuberculosis*,* chronic psychotic patients institutionalized in long-term care facilities are among high-risk groups for tuberculosis. Moreover, such settings might lack routine TB infection control practices and densely populated by persons with multiple risk factors for TB infection [11]. Studies consistently showed that a higher incidence of tuberculosis among patients with schizophrenia compared with the general population. The importance of screening for TB infection among individuals with different chronic psychotic disorders, especially those institutionalized in long-term care facilities is very important to control tuberculosis transmission [9].

Psychiatric service centers possess one or more potential risk factors for tuberculosis and other chronic diseases creating multiple burdens for psychotic patients. The multiplied burdens are due to different reasons, but the majority one is that psychotic disorders co-occurring with medical illnesses and some antipsychotic drugs such as clozapine which potentially make patients prone to tuberculosis. Co-morbidity of the diseases is also enabled by shared common risk factors such as, smoking, drug users, DM, alcoholism and HIV [12, 13].

Rates of smoking are markedly higher among people with psychotic illness than in the general population and some evidences show that smoking is directly related with mental illness and probably increase the risk of tuberculosis among psychotic patients. Active smoking was associated with an increased risk for developing active pulmonary TB in adults and number of cigarettes/day and duration of smoking are strongly associated with active pulmonary TB and tuberculosis-related mortality [13, 14].

Recreational drug use contributes for infection with *Mycobacterium tuberculosis* and the progression to active disease are both facilitated by a number of factors related to the risky lifestyle and behavior of such users which include; the crowded housing conditions; the accumulation and isolation of people indoors for the consumption of illicit drugs: poor ventilation of shelters or safe drug consumption rooms; the sharing of materials such as pipes and the expected malnutrition and severe cough presented by many users. There is adequate evidence linking drug and harmful substance use to modification of inflammatory responses in TB infection [15, 16].

Epidemiological and other evidence strongly supports the hypothesis that heavy alcohol use constitutes a risk factor for incidence of active tuberculosis and re-infection of TB. Prevalence of TB is higher among patients with alcohol-related disorders and it has been estimated that approximately 10% of all tuberculosis cases are attributable to alcohol use. Moreover, heavy drinking problem is causally related to the incidence and the worsening of the disease course [17, 18].

However, far too little attention has been paid to the incidence of tuberculosis in this psychiatric hospital and there is no integration of mental health service with screening of tuberculosis program implemented in Ethiopia. Currently there is no data on the prevalence of tuberculosis among people with severe mental illness and association of severe mental illness and tuberculosis at national level as well as facility level, so conducting this study in the area is insight for future studies in Ethiopia. The aim of this study was to assess the prevalence of active tuberculosis and associated risk factors among people with chronic psychotic disorders institutionalized at St. Amanuel mental specialized Hospital and Gergesenon Mental rehabilitation center.

## Methods and materials

### Study setting and period

The study was conducted at St. Amanuel Mental Specialized Hospital and Gergesenon Mental rehabilitation center from February to June, 2020, Addis Ababa, Ethiopia. Addis Ababa is the capital city of Ethiopia, established in 1887. According to the 2007 census, the city has a population of 2,739,551 inhabitants located in the geographic center of the country which lies at an altitude of 7546 feet (2300 m) with a grass land biome. Gergesenon is non-profit local mental rehabilitation association that provides support for patients collected from homeless with different type of mental illness in the center and provides care with trained psychiatry professionals. It is located around shiromeda area specific location of so called Kuskuam. On the other hand, St. Amanuel Hospital is a referral hospital that provides psychiatric specialty services to an average of 800 patients visit daily and has 270 beds including private wings for admission.

### Source population

All patients who were admitted at St. Amanuel Mental Specialized Hospital and Gergesenon Mental rehabilitation center for different chronic psychotic disorders.

### Study population

All patients who were admitted at St. Amanuel Mental Specialized Hospital and Gergesenon mental rehabilitation center for different chronic psychotic disorders during the 5 months of the study period.

### Study design

An institutional based cross-sectional study design was implemented to determine the prevalence of active tuberculosis and associated factors among chronic psychotic patients admitted at St. Amanuel Mental Specialized Hospital and Gergesenon Mental rehabilitation center.

### Inclusion criteria

All patients with ≥ 18 years old, who have been diagnosed with different mental health conditions and admitted in the health facility or mental health care facility during the data collection period.

### Exclusion criteria

Patients who are too ill and those who cannot talk or communicate were excluded.

### Operational definition

Active tuberculosis: Active tuberculosis refers to disease that occurs in someone infected with *M. tuberculosis complex bacteria.* It is characterized by signs or symptoms of active disease, or both, and is distinct from latent tuberculosis infection, which occurs without signs or symptoms of active disease [19]. Rifampicin resistance TB (RR-TB): Resistance to rifampicin detected using phenotypic or genotypic methods, with or without resistance to other anti-TB drugs. It includes any resistance to rifampicin, whether mono-resistance, multidrug resistance, polydrug resistance or extensive drug resistance [20].

Systematic screening for active TB: Systematic screening for active TB is the systematic identification of people with suspected active TB, in a pre-determined target group, using tests, examinations or other procedures that can be applied rapidly. Among those screened positive, the diagnosis needs to be established by one or several diagnostic tests and additional clinical assessments, which together have high accuracy [21].

Mental disorder: Syndrome characterized by clinically significant disturbance in individuals cognition, emotion regulation, or behavior that reflects a dysfunction in the psychological, biological or developmental process underlying mental functioning [22].

### Sample size and sampling technique

For this study, all admitted psychotic patients were screened for any sign of tuberculosis to identify presumptive TB cases. For logistics and time reason the sample size was estimated to enroll minimum representative presumptive TB cases using a single population proportion formula with the following assumption: Positivity rate of active tuberculosis among presumptive cases with mental health 16% [23], margin of sampling error tolerated 5% (0.05), at 95% confidence level of certainty (1.96), and 10% for non-response making the final sample size of 228.

### Data collection and processing

All illegible patients/attendants were requested for their verbal consent before asking any screening questions. Participants who gave their consent were screened for TB by using predefined TB screening criteria (2 or more weeks of cough and two or more of constitutional symptoms such as night sweating, fever and weight loss).

Participants who fulfilled the TB screening criteria were requested to give written consent in order to proceed for data collection. Consented participants were interviewed with predesigned questioner to collect some data on socio-demographic characteristics such as, residence, marital status, ethnicity, and educational status data, Behavioral risk factors such as smoking, alcohol drinking, Khat use, drug use, socioeconomic factors such us homelessness and clinical factors such us type of mental disorder, HIVsero-status and duration of admission. Additionally, 2 ml sputa sample was collected from each participant using recommended representative sample as follows.

Participants were instructed by trained professional psychiatry nurses to collect in a separate, ventilated room or preferably outdoors/produce sputum in sputum coughing designated area. Sputum specimens were collected in the early morning under the coaching of oriented staffs and patients were instructed on the right kind and volume of sputum to be collected. One sample of sputum was collected from each suspected patients in leak proof, screw cap with wide mouthed sputum cups and upon receipt of the specimens, proper labeling was verified before processing the specimens. The specimens were processed within 30 min of receipt at the laboratory.

## Xpert MTB/RIF test principle

### Xpert MTB/RIF test

Xpert MTB/RIF test is a semi-quantitative, nested real-time PCR diagnostic test for the detection of *M. tuberculosis* DNA in sputum samples. Additionally, it detects rifampicin resistance associated mutations of the rpoB gene within 81 bp RRDR (Rifampicin Resistance Determining Region) in samples from patients at risk for RIF resistance by using three unique primers and five specific hybridized probes. The Xpert MTB/RIF test is intended for use with specimens from untreated patients for whom there is clinical suspicion of tuberculosis, so that the results can be provided within 2 h.

### Xpert MTB/RIF procedure

All procedure followed from Xpert MTB/RIF manual and the basic procedures were collecting sputum sample from the patient with suspected TB then the sputum is mixed with the reagent (NALC-NaOH) that is provided with the assay for the purpose of decontamination and liquefied the sputum and lysing of cells. Cartridge containing this mixture was placed in the GeneXpert machine. All processing from this point on was fully automated from DNA extraction, amplification and detection.

### Data quality assurance

Site assessment and pre-test were done prior to data collection. All data quality control tools were considered. The data were cheeked for completeness and representativeness prior to entry. The GeneXpert machine was tested by Sample Processing Control (SPC) and Probe check control (PCC) for its performance. Pre-analytical, analytical and post-analytical stages of quality assurance that are incorporated in standard operating procedures (SOPs) of the microbiology laboratory of Saint Amanuel Mental Specialized Hospital was strictly followed. Generally, all laboratory analyses were carried out using standard operating procedures.

### Data analysis and interpretation

Data were presented into descriptive statics with frequency, proportion, percentages, measures of central tendency and standard deviation. Data were also entered and analyzed using the statistical software SPSS version 25. The Chi-square test was calculated to determine the association between variables, P-value of ≤ 0.05 was considered to test statistically significant association.

## Results

### Socio-demographic characteristics

A total of 3600 psychotic patients were admitted at the two rehabilitation and psychiatry centers during the data collection period. Of them, 60% (n = 2160) were admitted at St. Amanuel Mental Specialized Hospital and 40% (n = 1440) were admitted Gergesenon Mental Rehabilitation center.

A total of 250 presumptive TB cases were identified by using WHO recommended TB screening criteria. Sputum samples were tested by Xpert MTB/RIF assay. From these, 27 (10.8%) were positive for Xpert MTB/RIF assay.

The median and mean ± SD age of the presumptive TB cases were 35 and 36.5 ± 9.7 years respectively with the age ranged from 18 to 65 years. Furthermore, (68.4%) of the participants were male, nearly one third (32.4%) of the participants were within 34–41 years of age, more than half (52%) of the participants were single, one fifth (20.8%) of the participants were merchants, 67.7% of the participants were urban residents and 79.6% of the participants had formal education. Comparison of the sociodemographic characteristics of Xpert MTB/RIF positive and negative participants shows no significant difference (Table 1).

**Table 1:** Comparison of socio-demographic characteristics of Xpert MTB/RIF positive and negative presumptive TB cases among participants with chronic psychotic disorders, Addis Ababa, Ethiopia (February to June, 2020)

Variables/characteristics	Xpert MTB/RIF positive n (%)	Xpert MTB/RIF negative n (%)	Total n (%)	P-value
Sex				
Male	19 (11.1)	152 (88.9)	171 (68.4)	0.81
Female	8 (10.1)	71 (89.9)	79 (31.6)	
Age group (years)				
18–25	7 (16.7)	35 (83.3)	42 (16.8)	0.56
26–33	6 (10.3)	52 (89.7)	58 (23.2)	
34–41	7 (8.6)	74 (91.4)	81 (32.4)	
42–49	3 (7.1)	39 (92.9)	42 (16.8)	
Above 50	4 (14.8)	23 (85.2)	27 (10.8)	
Marital status				
Single	14 (10.8)	116 (89.2)	130 (52)	0.81
Married	10 (11.1)	80 (89.9)	90 (36)	
Widowed	2 (16.7)	10 (83.3)	12 (4.8)	
Divorced	1 (5.6)	17 (94.4)	18 (7.2)	
Occupation				
Health worker	0	7 (100)	7 (2.8)	0.91
Daily labor	4 (16.7)	20 (83.3)	24 (9.6)	
Merchant	6 (11.5)	46 (88.5)	52 (20.8)	
Student	3 (8.3)	33 (91.7)	36 (14.4)	
Farmer	7 (17.1)	34 (82.9)	41 (16.4)	
Others	7 (7.7)	83 (92.3)	90 (36)	
Residence				
Urban	17 (10.1)	152 (89.9)	169 (67.6)	0.66
Rural	9 (13.8)	56 (86.2)	65 (26)	
Homeless	1 (11.1)	8 (88.9)	9 (3.6)	
Jail	0	7 (100)	7 (2.8)	
Education				
Formal education	19 (9.5)	180 (90.5)	199 (79.6)	0.20
No formal education	8 (15.7)	43 (84.3)	51 (20.4)	

*n* number, *COR* crude odd ratio, *CI* confidence interval

### Prevalence of active tuberculosis and associated factors

The overall prevalence of confirmed pulmonary active tuberculosis was 750 per 100,000 population with chronic psychotic disorder.

To identify some risk factors associated with active TB among patients with chronic psychotic disorder, we compare the 27 Xpert MTB/RIF positive with 223 negative presumptive TB cases. Only the number of patients per single room was significantly associated with Xpert MTB/RIF positive active TB cases. The rest clinical and behavioral factors including, HIV, self-reported type II diabetes, smoking, alcoholism, Khat chewing, substance and drug use, type of psychotic disorder, and duration of institutionalization were not significantly associated with Xpert MTB/RIF positive active TB though percentage of frequency distribution seem different (Table 2).

**Table 2:** Association of clinical and behavioral factors with Xpert MTB/RIF positive active tuberculosis among participants with chronic psychotic disorders, Addis Ababa, Ethiopia (February to June, 2020) (n = 250 presumptive cases)

Variables/characteristics	Confirmed TB n (%)	Presumptive TB n (%)	Total n (%)	P-value
HIV status				
Positive	7 (15.6)	38 (84.4)	45 (18)	0.25
Negative	20 (9.8)	185 (90.2)	205 (82)	
Self-reported diabetes				
Diabetic mellitus	3 (12.5)	21 (87.5)	24 (9.6)	0.77
Non-diabetic mellitus	24 (10.6)	202 (89.4)	226 (90.4)	
Smoking				
Smokers	15 (9.8)	138 (90.2)	153 (61.2)	0.52
Non smokers	12 (12.4)	85 (87.6)	97 (38.8)	
Alcoholism				
Yes	13 (8.3)	143 (91.7)	156 (62.4)	0.10
No	14 (13.9)	80 (85.1)	94 (37.6)	
Khat				
Yes	14 (9.3)	137 (90.7)	151 (60.4)	0.33
No	13 (13.1)	86 (86.9)	99 (39.4)	
Substance				
Yes	14 (10.7)	117 (89.3)	131 (52.4)	0.95
No	13 (10.9)	106 (89.1)	119 (47.6)	
Illicit drug user				
Yes	8 (16.7)	40 (83.3)	48 (19.2)	0.14
No	19 (9.4)	183 (90.6)	202 (80.8)	
Mental illness				
Schizophrenia	18 (15.3)	100 (84.7)	118 (47.2)	NA
Bi-polar	7 (11.3)	55 (88.7)	62 (24.8)	
Depression	2 (5.3)	36 (94.7)	38 (15.2)	
Psychosis	0	32 (100)	32 (12.8)	
Duration of institutional ( month)				
< 1	2 (3.3)	58 (96.7)	60 (24)	0.157
1–2	9 (14.8)	52 (85.2)	61 (24.4)	
2–3	5 (10.2)	44 (89.8)	49 (19.6)	
> 3	11 (13.8)	69 (86.2)	80 (31.6)	
Number of patient in one ward				
13–20	7 (8.4)	76 (91.6)	83 (33.2)	0.038
21–28	14 (16.7)	70 (83.3)	84 (33.6)	
29–36	5 (15.6)	27 (84.4)	32 (12.6)	
> 37 patients	1 (2)	50 (98)	51 (20.4)	
1st symptom of mental illness begins				
Less than 1 year	5 (10.9)	41 (89.1)	46 (18.4)	0.51
2–3 years	7 (10.1)	62 (89.9)	69 (27.6)	
4–5 years	6 (7.7)	72 (92.3)	78 (31.2)	
> 6 years	9 (15.8)	48 (84.2)	57 (22.8)	

*n* number, *COR* crude odd ratio, *CI* confidence interval, *NA* not applicable

## Discussion

Tuberculosis remains a major public health problem worldwide and leading cause of morbidity and mortality. As described in the literatures, various factors could explain the high TB prevalence among mentally ill people. In Ethiopia previous studies have almost exclusively focused on estimating the magnitude of mood and psychotic disorders among patients following anti-TB treatment and HIV*-*co-infection. There was no single evidence in Ethiopia about the burden of TB among peoples with mood (depression and Bi-polar) disorders and psychotic (schizophrenic and non-Schizophrenic) disorders. Thus, the present study assessed the prevalence of active tuberculosis among these high risk special population in psychiatry centers (Gergesenon and St. Amanuel Mental Specialized Hospital) in Addis Ababa, Ethiopia.

In the current study, the prevalence of active tuberculosis was 750 per 100,000 population. This result is more than five times higher compared with the prevalence of TB in the general population of Ethiopia which is 140 per 100,000 population, 2020 [24]. The possible reason might be due to the observed poverty, substance abuse, homelessness, HIV, overcrowdings, malnutrition, smoking, alcoholism and poor seeking of health care behavior on mental illness in our study area.

The finding in the current study is comparable with a study report from New Guinea 765 per 100,000 [16], but lower than reports from Gondar 1482 per 100,000 [25], Ethiopia (prison facility), 1913 per 100,000 [26], Afghanistan 3567 per 100,000 [23]), Zambia 870 per 100,000 [27], China 985/100,000 population [28]. The prevalence of tuberculosis in the present study is also higher than from reports of Addis Ababa 189 per 100,000 [29], Ethiopia (prison facility) 458.1 per 100,000 [30], India 101.4 per 100,000 [31], Paraguay 41 per 100,000 population [32]. The possible reason for the difference might be associated with the variation in diagnostic methods used, geographical location associated with local prevalence, the socio cultural factors that could contribute for transmission of TB.

The observed prevalence of TB among mentally challenged populations in Addis Ababa, Ethiopia is unacceptably high. Given that Ethiopia has achieved remarkable success in meeting the previous stop TB partnership targets, sustainable development goals and on track to achieve end TB strategy goals [8]. One of the strategies of end TB goals is to address TB/HIV, multidrug-resistant TB and other challenged populations like peoples with mental health issues. We this regard, the finding from this study might be considered as a show case to show controlling TB in mental health is neglected. Controlling the burden of TB and achieving the 2035 end TB goals in such segment of the society is very far from reach.

The highest proportion of Xpert MTB/RIF positive active TB cases was detected from participant schizophrenia 18/250 (7.2%), this result was much higher as compared with as reports from different studies of similar case on the world, for instance; the report from Taiwan, 2018 showed people with schizophrenia had 0.88% [9]. The possible reason might be TB control programs is not implemented in our study area.

Likewise, finding in the current study was inconsistent as compared with some studies reported from different parts of the world, for example as a report from One meta-analysis conducted on 2013 from Greece showed that 2% (schizophrenia) from sanitarium, 3% (schizophrenia) from Nagasaki, Boston 20.2% (schizophrenia) psychiatry patients infected with tuberculosis [33]. The possible reason might be Ethiopia is categorized as high TB burden country and, in our study, we observed that the most common and deteriorative mental illness was schizophrenia that patients with this case were more likely infected by tuberculosis.

The current study also found that active tuberculosis was detected from diagnosed reason of Bi-polar 7/250 (2.8%) and Depression 2/250 (0.8%) with the presence of potential risk factors for tuberculosis. Few studies from Ethiopia reported that depression was the most common mental illness occurred following TB treatment on TB patients [34]. In our study statistically significant association was observed between chronic psychotic disorders and *Mycobacterium tuberculosis* infection.

Although we didn’t study the effect of medicines used to treat mental illness or the effect of TB drugs, the literature shows that Clozapine was the only antipsychotic agents associated with tuberculosis [9]. It is important that both psychiatrists and physicians are aware of the potential for interactions between the drugs used to treat tuberculosis and psychiatric conditions. Co-morbid mental illness is a significant unrecognized challenge for TB care and prevention in Ethiopia and this finding indicated that in Ethiopia no enough attention given to reduce the burden of TB in psychiatry centers.

In this study number of patients per room was significantly associated with Xpert MTB/RIF positive TB.

It is indicated in the result that average 25 individuals living in one room has the highest tuberculosis positivity rate detected in the study area. Increased household size and overcrowding have been documented as a risk factor for tuberculosis from several other studies in a variety of settings [35]. This is an important finding considering that inhalation is the major route of transmission of tuberculosis. Statistically significant association was observed between number of patients admitted in one room and active tuberculosis. This result may be explained by the fact that adult crowding reflects the increase likelihood of coming into contact with infectious persons excreting the bacilli in crowded environments.

## Conclusion

The prevalence of TB among peoples among patients with psychotic disorder is high. Overcrowding of patients during admission might contribute for the observed high prevalence of tuberculosis. These group are high risk population and prone to TB infection, disease and transmission. Despite this fact, controlling TB in peoples with psychotic disorder in Addis Ababa seems neglected. Thus, any government efforts targeted to control TB in mental health facilities should be intensified and consider the context of overcrowding. Considering TB control in mental health facilities should be considered as an integral part of national TB control strategy in Ethiopia.

## Data Availability

Data are available upon a request from the corresponding author.

## Abbreviations

AIDS: Acquired immune deficiency syndrome
CMD: Chronic mental disease
DM: Diabetes Mellitus
DOTS: Directly observed treatment and short course
DNA: Deoxyribose nucleic acid
HIV: Human immune virus
IRB: Institution Review Board
MDG: Millennium Development Goal
MDR: Multidrug- resistance
MTB: *Mycobacterium tuberculosis*
PCC: Probe check control
PCR: Polymerase chain reaction
RIF: Rifampicin resistance
SDG: Strategic Development Goal
SMI: Severe mental illness
SPC: Sample processing control
TB: Tuberculosis
TST: Tubercle Skin Test
WHO: World Health Organization
